# Short-Term Exercise Approaches on Menopausal Symptoms, Psychological Health, and Quality of Life in Postmenopausal Women

**DOI:** 10.1155/2010/274261

**Published:** 2010-08-16

**Authors:** Ayşegül Ağıl, Faruk Abıke, Arzu Daşkapan, Rıdvan Alaca, Handan Tüzün

**Affiliations:** ^1^Department of Physiotherapy, Bayındır Hospital, 06200 Ankara, Turkey; ^2^Department of Obstetrics & Gynecology, Bayındır Hospital, Sogutozu, 06200 Ankara, Turkey; ^3^Department of Physical Therapy, Başkent University Medical Faculty, 06710 Ankara, Turkey; ^4^Department of Physical Therapy, Bayındır Hospital, 06200 Ankara, Turkey

## Abstract

*Objective*. This study was designed to determine the effects of different short-term exercise programs on menopausal symptoms, psychological health, and quality of life in postmenopausal women. *Material and Methods*. Forty-two women were chosen from volunteering postmenopausal women presenting to the Department of Obstetrics and Gynecology of Bayındır Hospital between March and December 2009. The women aged 45–60 years and experiencing menopause naturally were included in the study. They were randomly divided into aerobic (*n* = 18) and resistance (*n* = 18) exercise groups. The women exercised 3 days per week for 8 weeks under the supervision of a physiotherapist. Aerobic exercise training was performed through a bicycle ergometer. Before and after the training, lipid profiles were measured and menopausal symptoms, psychological health, depression, and the quality of life were assessed through questionnaires. *Results*. In both exercise groups, no significant changes in lipid profiles were observed. In the resistance exercise group, excluding the urogenital complaints, there were significant improvements in all subscales of Menopausal Rating Scale (MRS). In the resistance exercise group, excluding the phobic anxiety, there were significant improvements in all subscales of The Symptom Checklist. Depression levels significantly decreased in both groups. Improvements were observed in all subscales of menopause-specific quality of life questionnaire in both groups except for sexual symptoms. *Conclusion*. Resistance exercise and aerobic exercise were found to have a positive impact on menopausal symptoms, psychological health, depression, and quality of life.

## 1. Introduction

Menopause is an adaptation process during which women go through a new biological state. This process is accompanied by many biological and psychosocial changes. During menopause, loss of skin flexibility, a decrease in libido, sexual dysfunction, an increase in the risk of cardiovascular diseases, urinary tract infections, incontinence, bone loss, and somatic and vasomotor symptoms may appear [1, 2]. Depressed mood, sleep disorders, and other psychological problems reduce the quality of life in postmenopausal women [3]. 

Due to the complexity of menopausal symptoms, many different alternatives to hormone replacement therapy have been developed to control menopausal symptoms. They include the use of herbal drugs, diet/nourishment, exercise programs, and lifestyle modification programs [4]. It is evident that exercise is becoming one of the most important alternative treatment procedures [3, 5]. There are studies in the literature that deal with the effect of exercise on menopausal symptoms. It is worth noting that in most of the studies aerobic and resistance exercises are used together [6]. It is also noteworthy that there are a limited number of studies that compare and contrast different types of exercise for postmenopausal women in terms of effectiveness. The aim of this study is to determine the effects of different short-term exercise programs on menopausal symptoms, psychological health, and quality of life in postmenopausal women and to evaluate the results in the light of the literature. 

## 2. Material and Methods

Forty-two women were chosen from volunteering postmenopausal women presenting to the Department of Obstetrics and Gynecology of Bayındır Hospital between March and December 2009. Written approval was obtained from the ethical committee. The women fulfilling the inclusion criteria were informed as to the purpose, method, content, usefulness, and duration of the study and were included in the study after they gave written consent. The women aged 45–60 years and experiencing menopause naturally were included in the study. The exclusion criteria were severe metabolic and endocrine diseases, receiving hormone replacement treatment, and surgical menopause. Furthermore, the patients taking selective estrogen receptor agonists (raloxifene), the patients receiving chemotherapy or radiotherapy for any cancer types, patients taking antidepressants or antipsychotics, patients unable to exercise, and those exercising regularly for the past 6 months were not included in the study. 

Prior to the study, 42 postmenopausal women were randomly divided into two different exercise groups: aerobic and resistance. Three cases from each group were unable to complete the study because of personal reasons. Data collected from these cases were not included in statistical analyses. Both groups attended the exercise programs 3 days per week for 8 weeks under the supervision of a physiotherapist. 

Before evaluation, ages, heights, and body mass indexes (BMI) of the cases were recorded. They were asked their professions, personal and family histories, educations and marital statuses, the ages when they went through menopause, their most serious health complaints, and medications they were using regularly. Before and after the study, of both groups blood lipid profiles and menopausal symptoms (Menopause Rating Scale-MRS), psychological symptoms (SCL-90-R The Symptom Checklist), depression (Beck Depression Inventory (BDI), quality of life regarding health evaluations; Menopause-specific Quality of Life Questionnaire (MENQOL) were carried out. MRS, MENQOL ve BDI questionnaires were done before the end of the study by only a physiotherapist as face to face and answer to question. It was taken blood samples of patients were taken before and after the study for lipid profile analyses. Lipid values of those included in the study were measured by using Beckman Coulter (SYNCHRON/Clinical system CX9 PRO) criteria. For evaluating menopausal symptoms, menopause symptoms evaluation scale was used [7]. Evaluation of psychological symptoms was carried out by using Symptom Scan List (SCL-90-R) [8]. Evaluation of depression was carried out by using Beck Depression Inventory [9, 10]. In evaluation of quality of life regarding health Menopause-specific Quality of Life Questionnaire (MENQOL) was used [11, 12]. 

Prior to aerobic exercise training, a submaximal and symptom-limited test on an ergonomic bike was carried out in patients with the aim of determining instructional workload. For the training, an ergonomic bike test was carried out in patients, ECG and a pulse controlled ergometric bike (Tunturi 460 ECG Pulse Controlled Ergometer) were used (Figure. Bike). Following warm up exercises, each case was trained on the bike for 40–45 minutes. Cases in the resistance exercise group were trained with elastic bands on big muscle groups. Prior to resistance exercise training, *perceived exertion scale* and *multimax repeated system* were used so as to determine the *elastic resistance regime* of cases [13, 14].

Exercises used in the resistance exercise (figure regime):

1. Standing chess press;
2. Strengthening back muscles in sitting position;
3. Standing strengthening abduction of both shoulders;
4. Standing strengthening flexion of both shoulders;
5. Standing strengthening flexion of both forearms;
6. Standing strengthening extension of both forearms;
7. Strengthening extension of both knees in sitting position;
8. Strengthening flexion of both knees in sitting position;
9. Strengthening dorsiflexion of both ankles in sitting position.

Data collected were analyzed by using SPSS edition 13.0. Whether or not data fit the normal distribution was evaluated by using Shapiro-Wilk test and distortion and perpendicularity coefficients. Fisher's Exact Chi-Square test or Chi-Square test was utilized with the aim of comparing two or more classes qualitative variables between groups. Comparison of data collected before and after the exercise programs, which are presented as average, in and among groups, was made by using, respectively, Wilcoxon signed-rank test and Mann-Whitney *U* test. Statistical significance level was accepted as <0.05.

## 3. Results

While the average age of cases in resistance exercise group was 52.6 ± 3.5 years, average age of cases in aerobic exercise group was 52.4 ± 4.7 years. Groups were similar to one another in terms of age, educational background, marital status and, job status (*P* >  .05). While the average age of those in resistance exercise group when they went through menopause age was 46.6 ± 4.5, it was 45.2 ± 4.1 in aerobic group. They were also similar to one another in terms of obstetrical features, first and last pregnancy ages, pregnancy, number of children, and the ages when they went through menopause (*P* >  .05). None of the cases in both groups had any habit of exercising (Table 1).

**Figure 1:**
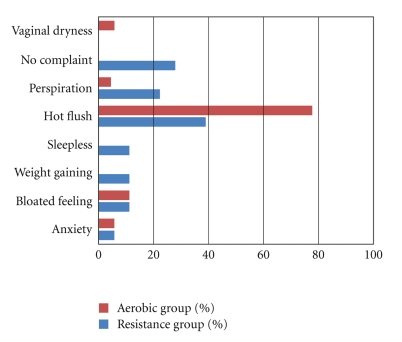
Most serious complaints of cases in resistance and aerobic exercise groups in postmenopausal period.

38,9% of cases in resistance exercise group and 77,8% of cases in aerobic exercise group stated that their most serious health complaint in postmenopausal period was “hot flush”. The rate of those whose most serious complaint in postmenopausal period was “perspiration” was 22,2% in resistance exercise group and 44,4% in aerobic exercise group. 27,8% of cases in resistance exercise group had no complaints pertaining to postmenopausal period (Figure 1). Prior to exercise programs, no statistically significant differences were found between the groups with respect to body mass index and waist hip ratio (*P* >  .05). The change ratio in both BMI and Waist-Hip Ratio(WHR) values of cases in aerobic exercise group obtained before and after the exercise programs was found to be not statistically significant (*P* >  .05). The change ratio in BMI values of cases in resistance group obtained before (26.7 ± 4.2 kg/m^2^) and after (26.3 ± 4.3 kg/m^2^) the program was found to be statistically significant (*P* <  .05). 

Before the program began, the groups were similar in terms of triglyceride, total cholesterol, High Density Lipoprotein (HDL), and Low Density Protein (LDL) values (*P*  >  .05). In triglyceride, total cholesterol, HDL, and LDL values of cases in aerobic exercise group, no statistically significant difference was detected compared to before (*P*  >  .05). The change in triglyceride values (155.9 ± 69.5 mg/dl; 106.4 ± 56.2 mg/dl) of cases in resistance exercise group compared to before was found to be statistically significant (*P*  <  .05) (Table 2). 

Before the exercise program began, the groups were similar in terms of MRS subscale points (*P*  >  .05). In MRS “Psychological complaints” and “somatic-vegetative complaints” subscale points and total points of cases in resistance exercise group, a statistically significant decrease was found when compared to before (*P*  <  .05). Yet, in “Urogenital complaints” subscale points, no statistically significant change was found when compared to before (*P*  >  .05) (Table 3).

**Figure 2:**
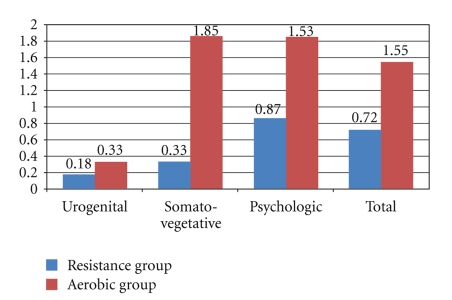
The Influence Quantity calculated for changes in subscales of MRS scale. IQ: Influence Quantity.

In MRS subscale points and total points of cases in aerobic exercise group, a statistically significant decrease was found when compared to before (*P*  <  .05) (Table 3). The influence quantity calculated for MRS scale subscale points of cases in aerobic exercise group was found to be higher than that of resistance exercise group (Figure 2). 

Before the exercise program began, groups were similar in terms of SCL-90 subscale and BDI points (*P* >  .05). In all SCL-90 R subscale points of cases in resistance exercise group except “phobic anxiety” subscale, a statistically significant decrease was found when compared to before (*P*  <  .05) (Table 4). In all SCL-90 R subscale points of cases in aerobic exercise group, a statistically significant decrease was found when compared to before (*P*  <  .05). The influence quantity calculated for changes in BDI points of resistance exercise group was found to be higher than that of aerobic exercise group (resistance IQ: 1,26; Aerobic IQ: 1,12) (Table 4).

Before the program began, groups were similar in terms of MENQOL scale subscale points (*P*  >  .05). In all MENQOL subscale points of cases in both groups except “Sexual Symptoms” subscale, a statistically significant decrease was found when compared to before (*P*  <  .05). The influence quantity calculated for changes in MENQOL subscale points of aerobic exercise group was found to be higher than that of resistance exercise group (Table 5). In the comparison of first and last values of exercise test parameters in aerobic exercise group cases, it was found that total test duration and workload reached had decreased statistically and significantly (*P*  <  .05) (Table 6).

## 4. Discussion

Physically active lifestyle can reduce the perceived intensity of menopausal symptoms and increase the state of being psychologically fine [15]. The fact that the medical staff dealing with women in menopausal period gets women adopt different habits and lifestyle in this critical period will be helpful in overcoming symptoms related to menopausal period. Recently it has been confirmed in the studies which evaluate the effects of exercise programs which contain aerobic and muscle strength training and exercise training programs accompanied by weight loss attempts that exercise has positive effects on the body composition of postmenopausal women [16, 17]. 

In the studies, it was found that short-term resistance exercise training in postmenopausal women does not make a significant change on BMI and waist hip ratio [18, 19]. In addition to the fact that in our study there was a slight decrease in BMI in both groups, this decrease in resistance exercise group reached the level of statistical significance. In terms of waist hip ratio, however, no significant change was observed in both exercise groups. It is reported that high levels of physical activity and particularly severe exercise are related to reduced body fat percentage and waist circumference [20]. It was considered that the intensity of exercise training being kept low and the duration of the training being short in aerobic exercise group might be an important reason why no positive effects of the exercise on the body composition was observed. In our study, BMI and waist hip ratio values were taken into consideration with the aim of evaluating the effects of aerobic and strength training on the body composition. 

It is well known that exercise reduces the risk of the development of coronary arterial disease in menopausal women and is effective on other health problems occurring frequently in menopausal period [21]. In postmenopausal women, as LDL and triglyceride levels increase, HDL level decreases [22]. In our study, it was observed that HDL, LDL, triglyceride, and total cholesterol levels of cases in aerobic group did not change significantly. In a study, it is stated that for a significant increase in HDL level to be obtained, a moderately intense activity which contains 16–24 km run for at least 4 months is needed [23]. According to another report, one should walk 3.2 km most days of the week in order to obtain the best health benefits from the activity [24]. According to the results of a comprehensive meta analysis, an exercise training that can affect the lipid levels of the exercise should last at least 12 weeks and be arranged in an intensity that it can provide weekly energy consumption (approx. 1200 cal.) [25]. Of cases in resistance exercise training, only in triglyceride values there was a significant decrease, but in other lipid parameters no significant change was observed. In our study, the fact that the duration of training being short (8 weeks)—each aerobic training session was approximately 30 min—and the intensity of the exercise being low was considered to explain why no positive effect on lipid profiles was observed.

In the exercise test done for aerobic exercise group after the training program, total test duration with maximum heart beat rate and workload increased. It was considered that in the light of these findings, short term low-intensity exercise training might increase the cardiovascular suitability of postmenopausal women.

It is stated in various studies and compilations in Cochrane database that physical activity and the participation in exercise have some significant positive effects on symptoms related to menopause [26]. The result of our study supported the idea that regular exercise has positive effects on somatic symptoms as stated in literature. In MRS points of cases in resistance and aerobic exercise groups after the training, significant decreases were observed. These decreases showed that the intensity of somatic complaints such as hot flush, cardiac problems, sleep disorders, and muscle-joint problems decreased in both postmenopausal women. This decrease in aerobic exercise group was found to be more significant than in resistance group in terms of influence quantity. According to MRS points after the training period, psychological symptoms of both groups had improved. After the training program, no significant change was observed in MRS points of postmenopausal women in resistance exercise group in terms of urogenital complaints. Although there were significantly improvements in MRS points of cases in aerobic exercise group in terms of urogenital complaints, it was noted that its influence quantity was low. It is emphasized that use of local low-dose estrogen is effective and safe in treatment of vaginal symptoms [27]. In our study, no special treatment or suggestion was made for urogenital complaints of cases in both groups.

At the end of our study, the effect of resistance and aerobic exercise trainings on menopausal symptoms was analyzed in general terms on the basis of changes in MRS. It was found that positive effects of aerobic exercise on menopausal symptoms were superior in terms of influence quantity.

It is emphasized that menopausal symptoms affect the quality of life in connection with their duration and intensity. Moriyama et al. showed that the quality of life of postmenopausal women who had bicycle training for 6 months in moderate intensity improved [28]. Ueda et al. obtained significant improvements in the quality of life after 12-week moderate aerobic exercise training [29]. In the study of Daley et al., it is stated that life scores related to health of women who had regular exercise more than 20 minutes, 3 days or more per week, in moderate intensity significantly increased when compared to those who did not [30]. MENQOL scale used in our study allows for evaluation of menopausal symptoms, associating them with the quality of life. Significant changes in all subscale points of the scale expect for sexual symptoms showed that both exercise programs increased the quality of life of the cases. It was found that aerobic exercise was slightly more effective in terms of influence quantity. It was concluded that the increase obtained in the quality of life might be parallel to improvement observed in menopausal symptoms of both groups. No studies that compare and contrast the effects of aerobic and resistance trainings on menopausal symptoms in postmenopausal women were found. However, the results we obtained support the studies that show the positive effects of exercise on the quality of life [28, 29]. 

In some studies it is stated that exercise has effect on psychological health of old women having appetite, weight, motivation loss, and low energy level. However, it is worth noting that the effect of exercise on health postmenopausal women is not mentioned [31, 32]. On the basis of this knowledge, psychological effects of two different exercise programs on women in postmenopausal period were analyzed in our study. Significant decreases in all subscale points of psychological symptoms scan list of cases in both groups showed the positive effects of both resistance and aerobic exercise training on psychological health of postmenopausal women. It was noted that psychological benefit observed in aerobic exercise group was slightly more evident. According to the results of epidemiologic studies, approximately 8% and 47% of women who have gone through menopause suffer from depression symptoms [33]. In a study that deals with postmenopausal women, it was shown that depression symptoms are seen more in women suffering from sleep disorders, and similarly there is a correlation between vasomotor and depression symptoms [34]. Asbury and his friends (Asbury et al.) reported that quality of life and depression level of postmenopausal women improved after 12-week aerobic exercise training [35]. The results related to depression of our study are in concordance with studies in literature. It was found that depression values of cases who had both aerobic and resistance training decreased significantly. 

It was seen that both resistance and aerobic exercise training have similar positive effects on menopausal symptoms, psychological health and quality of life, however aerobic exercise is slightly more effective in terms of influence quantity. The thing that matters more than getting postmenopausal women to participate in physical activity programs is to motivate them to do regular exercise throughout their lives. It becomes difficult for women in menopausal period to accommodate themselves to exercise because of the presence of some symptoms. For the exercise to be able maintain long-term health benefits, it has to be planned depending on the needs, preferences, and limitations of women particularly in relation with musculoskeletal system and cardiovascular suitability. In this sense, an active lifestyle should be established among women in postmenopausal period and suitable exercise programs should be designed. Our results are considered to be promising for studies that will be planned in the future with the aim of evaluating the effects of different exercise trainings on postmenopausal women.

In the resistance exercise group, excluding the urogenital complaints, there were significant improvements in all subscales of Menopausal Rating Scale (MRS). In the resistance exercise group, excluding the phobic anxiety, there were significant improvements in all subscales of The Symptom Checklist. Depression levels significantly decreased in both groups. Improvements were observed in all subscales of Menopause-Specific Quality of Life questionnaire in both groups except for sexual symptoms. Resistance exercise and aerobic exercise were found to have a positive impact on menopausal symptoms, psychological health, depression and, quality of life.

## Figures and Tables

**Table 1:** Demographics of groups.

Demographics	Exercise groups		*P*
	Resistance	Aerobic	
	(*N* = 18)	(*N* = 18)	
Age, *X* ± SD, years	52.6 ± 3.5	52.4 ± 4.7	.751^†^
Marital status, *n *(%)			
Married	14 (77.8)	14 (77.8)	
Single	—	2 (11.1)	.411^‡^
Divorced	4 (22.2)	2 (11.1)	

Smoking Habitus, *n *(%)			
Smoker	9 (50.0)	7 (38.9)	.502^‡^
Non-Smoker	9 (50.0)	11 (61.1)	
Alcohol, *n*(%)			
Drinker	4 (22.2)	8 (44.4)	.157^‡^
Non-Drinker	14 (77.8)	10 (55.6)	
Exercise Habit, *n *(%)			
Yes	—	—	NA
No	18 (100.0)	18 (100.0)	

**Table 2:** Lipid profile in groups.

	Into Resistance Before	Into Resistance After	*P* ^†^	Into Aerobic before	Into Aerobic after	*P* ^†^
Triglyceride	155.9 ± 69.5	106.4 ± 56.2	**.006**	126.9 ± 42.3	126.5 ± 43.9	.383
Total Cholesterol	238.9 ± 50.5	212.2 ± 42.9	.053	218.7 ± 26.1	226.9 ± 30.2	.210
HDL	53.4 ± 10.3	53.8 ± 10.9	.419	58.6 ± 13.5	60.7 ± 17.2	.305
LDL	153.9 ± 38.1	136.3 ± 37.4	.071	134.3 ± 23.1	140.6 ± 31.0	.286

**Table 3:** MRS scale subscale points of cases in resistance group before and after the program *X* ± SD, *N* = 18.

Subscales of Menopausal Symptoms Evaluation Scale	Before resistance exercise program	After resistance exercise program	*P* Value^†^	Before aerobic exercise program	After aerobic exercise program	*P* Value^†^
Psychological complaints	5.9 ± 3.4	3.5 ± 2.1	**.001**	7.6 ± 3.8	3.0 ± 2.2	**.001**
Somatic-vegetative complaints	6.1 ± 3.3	5.0 ± 3.4	**.015**	7.2 ± 3.7	2.3 ± 1.6	**.001**
Urogenital complaints	2.2 ± 1.7	1.9 ± 1.6	.197	3.6 ± 2.9	2.7 ± 2.5	**.016**
Total	14.2 ± 6.2	10.4 ± 4.4	**.001**	18.3 ± 8.3	8.1 ± 4.9	**.001**

^†^: Wilcoxon signed-rank test.

**Table 4:** SCL-90-R subscale and BDI points of Aerobic and Resistance exercise groups before and after the exercise program *X* ± SD, *N* = 18.

SCL-90-R Subscales-BDI	Before resistance exercise program	After resistance exercise program	*P* Value^†^	IQ	Before Aerobic exercise program	After aerobic exercise program	*P* Value^†^	IQ
Somatization	1.06 ± 0.7	0.61 ± 0.5	**.001**	0.75	1.09 ± 0.9	0.56 ± 0.5	**.001**	0.76
Anxiety	0.61 ± 0.5	0.39 ± 0.4	**.001**	0.49	0.71 ± 0.5	0.38 ± 0.4	**.001**	0.73
Obsessive-Compulsive Dimension	0.87 ± 0.6	0.59 ± 0.5	**.001**	0.51	1.26 ± 0.7	0.74 ± 0.5	**.001**	0.87
Depression	0.77 ± 0.6	0.49 ± 0.5	**.001**	0.51	0.97 ± 0.8	0.56 ± 0.4	**.001**	0.68
Interpersonal sensitivity	0.50 ± 0.4	0.33 ± 0.4	**.005**	0.43	0.81 ± 0.6	0.50 ± 0.4	**.001**	0.62
Psychoticism	0.25 ± 0.3	0.19 ± 0.3	**.039**	0.20	0.59 ± 0.5	0.32 ± 0.3	**.001**	0.68
Paranoid Thoughts	0.52 ± 0.5	0.41 ± 0.4	**.011**	0.24	0.80 ± 0.5	0.45 ± 0.4	**.002**	0.78
Anger-Hostility	0.42 ± 0.4	0.28 ± 0.4	**.007**	0.35	0.63 ± 0.6	0.30 ± 0.3	**.003**	0.73
Phobic anxiety	0.39 ± 0.5	0.32 ± 0.4	.068	0.16	0.31 ± 0.4	0.18 ± 0.2	**.029**	0.43

Additional Scale	0.70 ± 0.6	0.55 ± 0.6	**.008**	0.25	1.24 ± 0.8	0.55 ± 0.4	**.001**	1.15

Average General Symptoms	0.64 ± 0.4	0.43 ± 0.4	**.001**	0.53	0.86 ± 0.6	0.44 ± 0.3	**.001**	0.93

Beck Depression Inventory	10.7 ± 5.8	5.1 ± 3.1	**.001**	1.26	11.9 ± 8.5	4.7 ± 4.4	**.001**	1.12

IQ: Influence Quantity, BDI: Beck Depression Inventory, ^†^Wilcoxon signed-rank test.

**Table 5:** MENQOL scale subscale points of Aerobic and Resistance exercise groups before and after the exercise program *X* ± SD, *N* = 18.

MENQOL scale subscales	Before resistance exercise program	After resistance exercise program	*P* Value^†^	IQ	Before aerobic exercise program	After aerobic exercise program	*P* Value^†^	IQ
Vasomotor symptoms	11.3 ± 5.9	10.5 ± 5.6	**.006**	**0.14**	14.2 ± 5.4	11.3 ± 12.7	**.005**	**0.32**
Psychosocial symptoms	22.6 ± 9.5	17.8 ± 7.5	**.002**	**0.56**	29.3 ± 12.0	20.7 ± 8.8	**.001**	**0.83**
Physical symptoms	55.7 ± 18.6	46.8 ± 17.5	**.004**	**0.49**	64.2 ± 15.6	52.7 ± 13.7	**.001**	**0.78**
Sexual symptoms	10.1 ± 7.0	10.1 ± 7.0	1.000	**0**	10.2 ± 7.9	9.7 ± 7.6	.088	**0.06**

IQ: Influence Quantity, ^†^: Wilcoxon signed-rank test.

**Table 6:** Exercise test parameters in aerobic group.

Exercise test parameters	First test	Last test	*P* value^†^
Total test into Duration, sec.	890.0 ± 168.8	1110.0 ± 224.7	**.001**
Workload reached, watt.	64.4 ± 9.4	76.7 ± 12.5	**.001**
Heart rate recovery, pulse/min.	85.8 ± 13.0	84.8 ± 11.2	.432
Diastolic blood pressure recovery, mmHg	7.0 ± 1.3	6.8 ± 1.6	.388
Systolic blood pressure recovery, mmHg	11.5 ± 1.9	11.2 ± 1.6	.509

^†^: Wilcoxon signed-rank test.
